# Photolysis of the Insensitive Explosive 1,3,5-Triamino-2,4,6-trinitrobenzene (TATB)

**DOI:** 10.3390/molecules27010214

**Published:** 2021-12-30

**Authors:** Annamaria Halasz, Jalal Hawari, Nancy N. Perreault

**Affiliations:** 1 National Research Council Canada, 6100 Royalmount Ave, Montreal, QC H4P 2R2, Canada; peca.annamaria@gmail.com; 2 Department of Civil, Geological and Mining Engineering, École Polytechnique de Montréal, Montreal, QC H3C 3A7, Canada; jalal.hawari@polymtl.ca

**Keywords:** energetic material, photolysis, sunlight simulation, hydrolysis, environmental fate

## Abstract

The explosive 1,3,5-triamino-2,4,6-trinitrobenzene (TATB) is of particular interest due to its extreme insensitivity to impact, shock and heat, while providing a good detonation velocity. To determine its fate under environmental conditions, TATB powder was irradiated with simulated sunlight and, in water, under UV light at 254 nm. The hydrolysis of particles submerged in neutral and alkaline solutions was also examined. We found that, by changing experimental conditions (e.g., light source, and mass and physical state of TATB), the intermediates and final products were slightly different. Mono-benzofurazan was the major transformation product in both irradiation systems. Two minor transformation products, the *aci*-nitro form of TATB and 3,5-diamino-2,4,6-trinitrophenol, were detected under solar light, while 1,3,5-triamino-2-nitroso-4,6-dinitrobenzene, 1,3,5-triamino-2,4-dinitrobenzene and mono-benzofuroxan were produced under UV light. The product identified as 3,5-diamino-2,4,6-trinitrophenol was identical to the one formed in the dark under alkaline conditions (pH 13) and in water incubated at either 50 °C or aged at ambient conditions. Interestingly, when only a few milligrams of TATB were irradiated with simulated sunlight, the *aci*-isomer and mono-benzofurazan derivative were detected; however, the hydrolysis product 3,5-diamino-2,4,6-trinitrophenol formed only much later in the absence of light. This suggests that the water released from TATB to form mono-benzofurazan was trapped in the interstitial space between the TATB layers and slowly hydrolyzed the relatively stable *aci*-nitro intermediate to 3,5-diamino-2,4,6-trinitrophenol. This environmentally relevant discovery provides data on the fate of TATB in surface environments exposed to sunlight, which can transform the insoluble substrate into more soluble and corrosive derivatives, such as 3,5-diamino-2,4,6-trinitrophenol, and that some hydrolytic transformation can continue even without light.

## 1. Introduction

For several decades, 2,4,6-trinitrotoluene (TNT), hexahydro-1,3,5-trinitro-1,3,5-triazine (RDX), and octahydro-1,3,5,7-tetranitro-1,3,5,7-tetrazocine (HMX) have been the most commonly used military explosives. However, to reduce the risk of accidental detonation, alternative energetic materials (EMs) with lower sensitivity to shock and impact are gradually replacing these compounds in explosive formulations. 1,3,5-Triamino-2,4,6-trinitrobenzene (TATB) is of particular interest as an EM due to its extreme insensitivity to impact, shock, and heat, while at the same time offering a good detonation velocity [1]. The stability of TATB is mostly due to its significant inter- and intramolecular hydrogen bonding, while other intermolecular interactions also make contributions [2].

The environmental fate of insensitive EMs, such as TATB, and their products requires particular attention because some insensitive munition rounds are less efficient in high-order and blow-in-place detonations, depositing greater masses of energetic residues per round [3]. The thermal decomposition of TATB has however recently been reported as showing the distribution of, principally, gaseous products [4]. Direct photolysis and hydrolysis are important abiotic processes that determine the fate of a chemical in the environment. Some investigators have studied the photo-induced decomposition of TATB [5,6,7,8] but the degradation mechanism(s), especially the key initial steps involved in the decomposition process, remain unclear and appear complex in nature, depending on the experimental conditions and methods used [8]. TATB was found to be photosensitive and changed color from yellow to green and even to dark colors when irradiated with sunlight, ultra-violet light, X-rays or gamma rays [5,6,7,8,9]. Attempts to identity the TATB green product have led to different assumptions, and the mechanism of color formation and transformation is still controversial. Williams et al. [7] confirmed the formation of a free radical using electron paramagnetic resonance (EPR) spectroscopy of the green powder generated after UV-irradiation of TATB. The free radical was later identified as a phenoxyl radical R–_NO_ [8]. Alternatively, the green color was attributed to the formation of the mono-nitroso derivative of TATB [6] or to a furazan derivative detected during gamma irradiation of TATB [9]. More recently, the color change of TATB was attributed to physical structural transformations instead [10].

The present study was undertaken to elucidate the long-lasting debate on the products distribution and photodegradation pathway of TATB and to gain more insight into its fate in the upper layer of soil and water bodies. TATB being practically insoluble in water, photolysis was studied as dry particles irradiated with artificial sunlight and as particles in water irradiated with UV at 254 nm in a Rayonet photoreactor. The stability of TATB in aqueous systems under different alkaline conditions and aged in water at ambient conditions was also investigated.

## 2. Results

### 2.1. Degradation of Dry TATB with Simulated Sunlight

**Figure 1:**
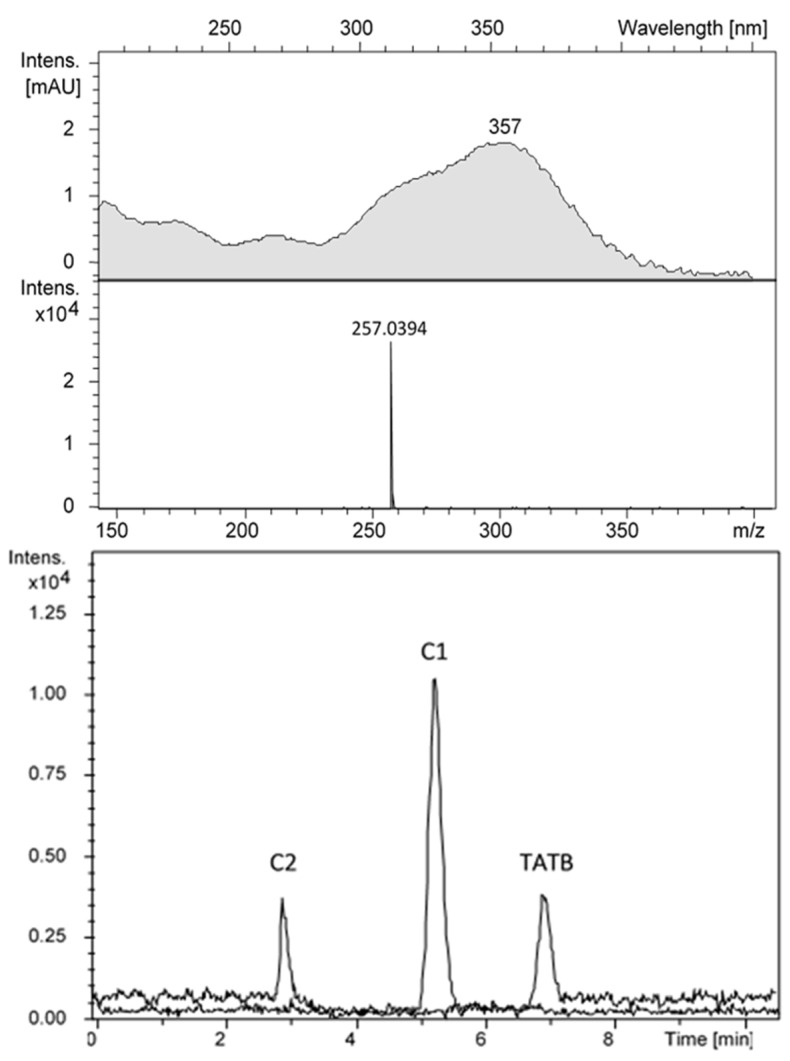
(**top**) UV and MS [M − H]^−^ spectra of TATB standard (0.273 mg L^−1^ in 50% acetonitrile in water); (**bottom**) extracted ion chromatograms (EICs) at *m*/*z* 239 (C1) and *m*/*z* 257 (C2 and TATB) of DMSO extract of TATB (2.1 mg) photolyzed in a SolSim photoreactor (280–800 nm) for 4 days.

Strong intramolecular hydrogen bonding is thought to result in the poor water solubility of TATB. The solubility of TATB in water was determined at 30 °C and found to be 0.01 mg L^−1^. Due to its very low solubility, photolysis was studied in the form of dry particles. Solvent-free TATB particles (2.5 ± 0.7 mg) were irradiated with simulated sunlight in a SolSim photoreactor. After 4 days of irradiation, the yellow particles turned dark green, similar to what had been previously reported [5]. No relevant change in product color and product distribution was observed when the samples were further irradiated for 6 days. The degradation products were analyzed by LC-MS using electrospray ionization in the negative mode (ESI-), producing mainly deprotonated molecular mass ions [M − H]^−^. Figure 1 (top) shows the UV and MS [M − H]^−^ spectra of TATB standard (0.273 mg L^−1^) in 50% acetonitrile in water (MeCN-50).

An LC-MS analysis of the photolyzed samples, extracted with DMSO, showed a major degradation product (C1) with a (M – H)^−^ at *m*/*z* 239.0316 Da matching an empirical formula of C_6_H_4_N_6_O_5_. Compound C1 was tentatively identified as the mono-benzofurazan derivative of TATB (Figure 1, bottom, and Table 1), similar to the compound detected using thermal decomposition under pressure [11] or gamma irradiation [9].

The compound eluting at 2.9 min (Figure 1, C2) showed a mass spectrum identical to TATB with a (M – H)^−^ at *m*/*z* 257.0276 Da matching an empirical formula of C_6_H_6_N_6_O_6_. We tentatively identified C2 as an aci-nitro isomer (tautomer) of TATB (Table 1). We suspect that one of these two products, C1 or C2, is the green-colored compound observed during the photolysis of TATB in a SolSim photoreactor.

When an increased amount of TATB (50 ± 5 mg) was photolyzed under the same conditions, a lighter green coloration was observed after 4 days and the samples were further irradiated for 6 days with no color intensification. An LC-MS analysis of the products extracted with DMSO or acetonitrile/water 50:50 (*v*/*v*) (MeCN-50) confirmed the predominant production of the mono-benzofurazan derivative (C1) and the presumed aci-nitro compound (C2) in trace amounts. The green product therefore appears to correlate with the formation of C1, i.e., the mono-benzofurazan derivative rather than the aci-nitro isomer of TATB. UV (200–450 nm) and MS spectra of compound C1 are presented in Figure S1.

**Figure 2:**
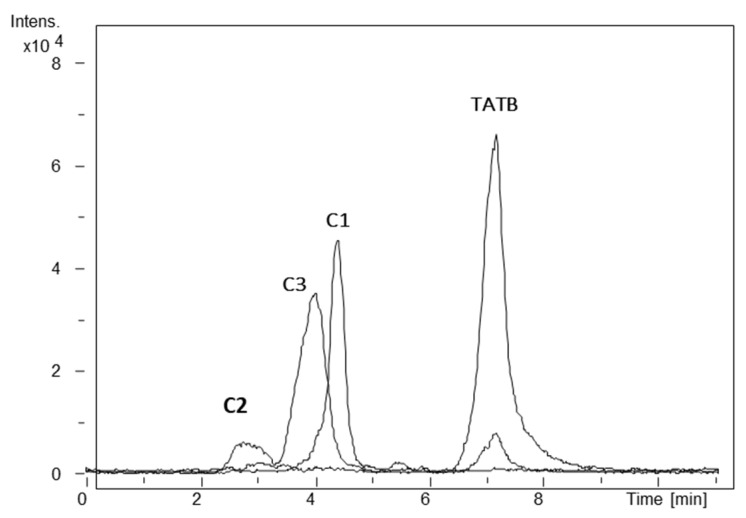
Extracted ion chromatograms (EICs) at *m*/*z* 239 (C1), *m*/*z* 257 (C2 and TATB), and *m*/*z* 258 (C3) of the acetonitrile extracts of TATB (54 mg) photolyzed in a SolSim photoreactor for 6 days.

In addition, we detected another compound, C3, with a (M − H)^−^ at *m*/*z* 258.0116 Da (Figure 2), matching an empirical formula of C_6_H_5_N_5_O_7_, which was identified by high resolution mass spectrometry to be 3,5-diamino-2,4,6-trinitrophenol (C3, Table 1).

**Figure 3:**
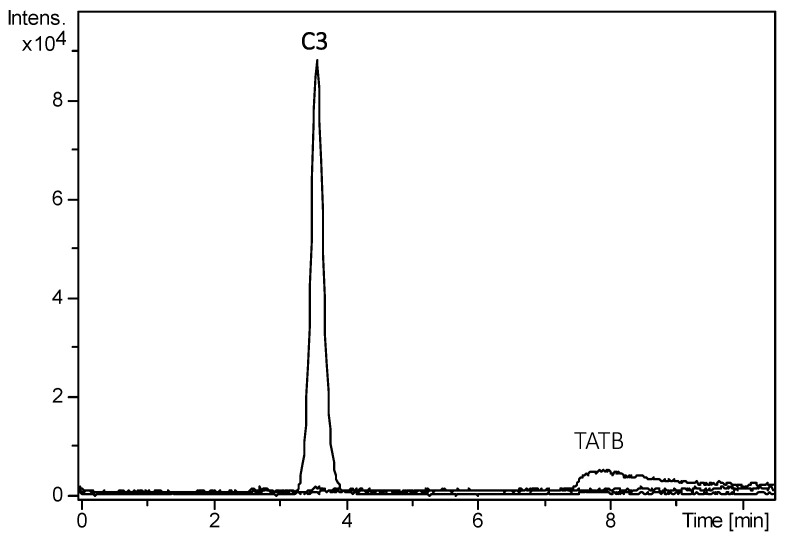
Extracted ion chromatograms (EICs) at *m*/*z* 257 (TATB) and *m*/*z* 258 (C3) of TATB in water incubated at 50 °C for 8 days.

We also detected C3 when the TATB particles in water were kept in the dark at 50 °C for 8 days (Figure 3) or aged at ambient conditions for 9 months. Furthermore, we detected C3 with two other products, namely 1,3-dihydroxy-5-amino-trinitrobenzene and 1,3,5-trihydroxy-2,4,6-trinitrobenzene, identified by LC-MS, when TATB was hydrolyzed under strong alkaline conditions (pH 13, 25 °C, 8 days). The color of the TATB particles did not change during the hydrolysis experiments, and the solutions gradually turned orange over time (data not shown). Thus, the presence of yellow-orange diamino-trinitrophenol derivatives in the photolyzed samples may explain the alteration from green to light green color described above.

To confirm the role of water in the formation of C3, TATB (54 mg) was photolyzed in a SolSim photoreactor for 4 days, then a few mg were mixed with 1 mL of acetonitrile and H_2_^18^O (1:1 *v*/*v*), held for 1 h and the supernatant was analyzed by LC-MS. The results showed the presence of a mass ion at *m*/*z* 260, an increase of 2 amu (atomic mass units) over that of C3, indicating the incorporation of one labeled-^18^O atom from H_2_^18^O with a yield of 7.9%.

**Figure 4:**
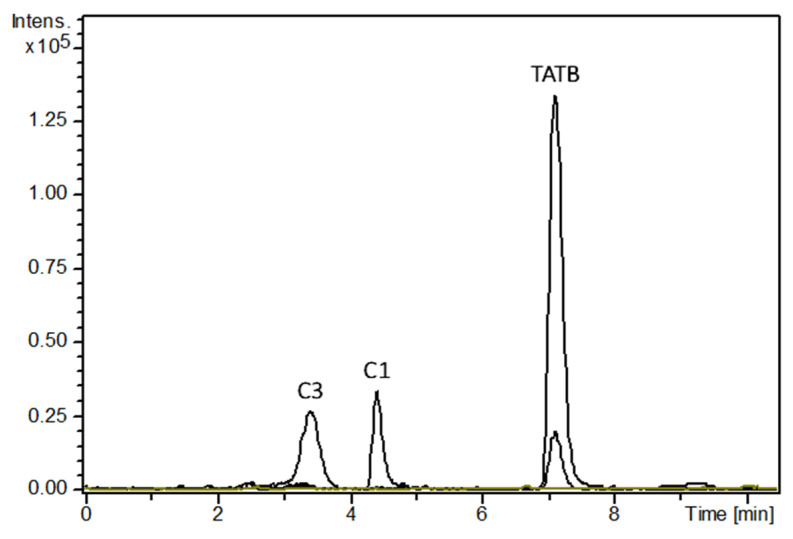
Extracted ion chromatograms (EICs) at *m*/*z* 239 (C1), *m*/*z* 257 (TATB) and *m*/*z* 258 Da (C3) of DMSO extract of TATB (2.5 mg) photolyzed in a SolSim photoreactor for 4 days and analyzed 6 months later, showing the absence of the *aci*-nitro isomer of TATB.

When the 2.5 mg dry TATB sample photolyzed in a SolSim photoreactor for 4 days was reanalyzed after being kept standing for 6 months at room temperature in the dark, the compound C3 was detected (Figure 4), but the product C2 that was observed in the sample of freshly prepared TATB (Figure 1) was not there. Furthermore, the relative abundance of TATB increased substantially in the sample stored for 6 months, as shown in (Figure 4). These experimental findings suggest that C2 was partially transformed into C3 and that their large parts, in turn, reconverted into TATB.

### 2.2. Degradation of TATB with UV at λ = 254 nm

**Figure 5:**
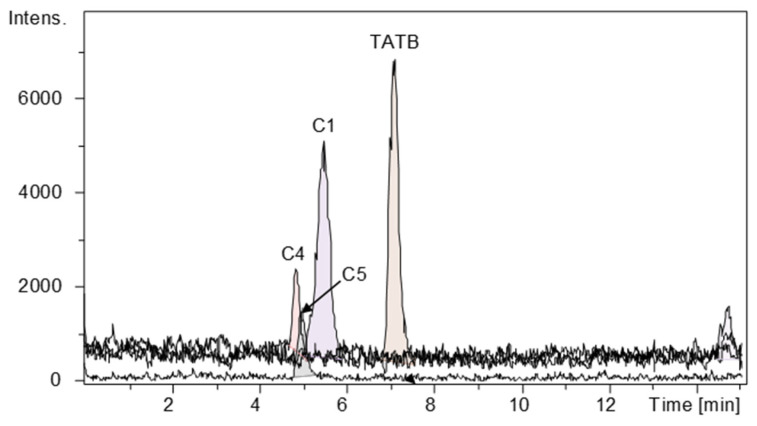
Extracted ions chromatograms (EICs) at *m*/*z* 241 (C4), *m*/*z* 212 (C5), *m*/*z* 239 (C1) and *m*/*z* 257 (TATB) of the sample (0.7 mg L^−1^) prepared in MeCN-50 (1:1 (*v*/*v*) mixture of water and acetonitrile) and photolyzed at 254 nm for 10 min.

The photodegradation of TATB (0.7 mg L^−1^) in 50% (*v*/*v*) of acetonitrile in water (MeCN-50) was completed in 30 min at λ = 254 nm. More than 80% TATB was lost after 10 min of irradiation. The disappearance of TATB was accompanied by the production of several products identified by their [M − H]^−^ as C1 (at *m*/*z* 239.0277 Da), C4 (*m*/*z* 241.0432 Da) and C5 (*m*/*z* 212.0528 Da), as shown in Figure 5.

The compounds of C4, with *m*/*z* 241 Da matching an empirical formula of C_6_H_6_N_6_O_5_, and C5, with *m*/*z* 212 Da matching an empirical formula of C_6_H_7_N_5_O_4_, were tentatively identified as the 1,3,5-triamino-2-nitroso-4,6-dinitrobenzene (C4) and 1,3,5-triamino-2,4-dinitrobenzene (C5) derivatives of TATB (Table 1). 

When excess TATB (10 ± 2 mg) equilibrated in water or in MeCN-50 (5 mL) was photolyzed for 24 h at 254 nm, the yellow TATB particles turned green, as observed for the dry particles irradiated with simulated sunlight. Mono-benzofurazan (C1) and 1,3,5-triamino-2-nitroso-4,6-dinitrobenzene (C4) were detected in trace amounts by LC-MS in the liquid phase. To enhance the solubilization of the compounds, the photolyzed samples were subjected to ultrasonic treatment (35 kHz) for 10 min and the liquid phase analyzed by LC-MS. Compound C1 appeared in great abundance in the liquids, suggesting that the reactions had occurred on the surface of the particles. Interestingly, when the sonicated samples were photolyzed for an additional 2 h, another compound (C6) appeared with [M − H]^−^ at *m*/*z* 255.0237 Da, representing an empirical formula of C_6_H_4_N_6_O_6_. The compound C6 was tentatively identified as a mono-benzofuroxan derivative of TATB (Table 1). C6 was only observed in saturated sonicated/photolyzed samples, suggesting that photolysis coupled with sonication resulted in some structural changes in TATB, initiating new degradation pathways.

## 3. Discussion

The identity of the photo-products of TATB and their appearance and disappearance were used to elucidate the initial decomposition routes of TATB. In our study, the mono-benzofurazan derivative (C1) was the predominant photo-product during the photolysis of TATB with simulated sunlight. Product C2, with a (M – H) similar to that of TATB (*m*/*z* 257 Da), was tentatively identified as an *aci*-nitro isomer of TATB. Nitro↔*aci*-nitro tautomerism is a common mechanism experienced by several *ortho*-substituted aromatic nitro compounds during photolysis [12]. Although *aci*-nitro compounds are considered to be very reactive, they become more stable through their ability to establish inter- and intramolecular hydrogen bonding due to their pertinent functionalities, i.e., NO_2_ and NH_2_ [13]. 

**Scheme 1:**
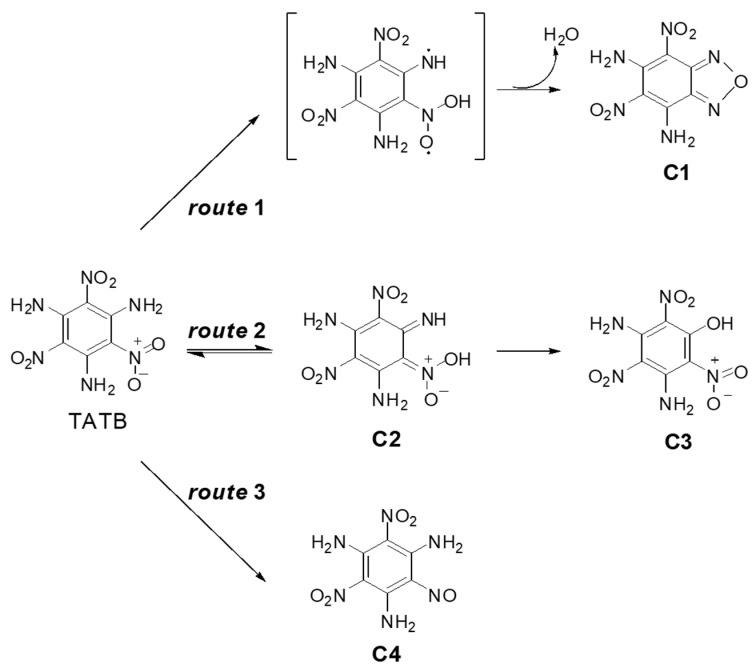
Postulated major (**1**) initial route involved in the photodegradation of TATB under both simulated sunlight and UV at 254 nm. The structure of compound in bracket was originally proposed by Wu and Fried [15]. Two minor routes, (**2**) and (**3**), were observed only under simulated sunlight and UV at 254 nm, respectively.

By browsing previous research, two plausible chemical process were proposed via a precursor to form the mono-benzofurazan derivative of TATB: (1) the formation of a biradical [11] and (2) nitro↔*aci*-nitro tautomerism [14]. The formation of a biradical mediated by intramolecular hydrogen transfer from the -NH_2_ to the -NO_2_ group has been suggested as a precursor to decomposition products such as benzofurazans with an activation barrier calculated to be 47.5 kcal/mol [15]. Free radical formation detected by EPR during UV-induced degradation of TATB has also been reported [7]. Although the formation of the *aci*-nitro isomer of TATB requires a relatively low energy of 42.1 kcal/mol, subsequent cyclization by removing water to produce mono-benzofurazan requires an overall activating barrier of 68.6 kcal/mol [14], which is much higher than that calculated for the biradical process, 47.5 kcal/mol [15]; this suggests that the precursor of the mono-benzofurazan derivative of TATB (C1) is rather a biradical during photolysis by simulated sunlight (Scheme 1, route 1).

The *aci*-nitro isomer of TATB (C2) slowly hydrolyzed to 3,5-diamino-2,4,6-trinitrophenol (C3) in the presence of water or returned to the nitro form when the light was off (Scheme 1, route 2).

The production of C3 (3,5-diamino-2,4,6-trinitrophenol) during the photolysis of dry particles of TATB can be attributed to the water produced during the formation of mono-benzofurazan and trapped in the interstitial space in the crystal structure of TATB [16]. We hypothesize that during the photolysis of a relatively large amount of TATB (50 to 60 mg), water was produced in sufficient amounts to initiate the hydrolysis of a moderately stable intermediate (C2) to produce C3 (3,5-diamino-2,4,6-trinitrophenol) (Scheme 1, route 2). The detection of ^18^O from H_2_^18^O in C3 is further evidence supporting the involvement of water in the formation of 3,5-diamino-2,4,6-trinitrophenol.

The detection of C3 from TATB in water held at 50 °C for 8 days, and even aged for 9 months at ambient conditions protected from light, suggests that the insoluble TATB will hydrolyze under natural environmental conditions into 3,5-diamino-2,4,6-trinitrophenol (C3), a seemingly more soluble compound (Figure 3). The hydrolysis of TATB can be accelerated under alkaline conditions (pH 13 at 25 °C). After 8 days of treatment, the production of 3,5-diamino-2,4,6-trinitrophenol (C3) was confirmed and two more hydrolysis products, 1,3-dihydroxy-5-amino-2,4,6-trinitrobenzene and 1,3,5-trihydroxy-2,4,6-trinitrobenzene (THTNB), have been identified by LC-MS. The transformation of TATB into water-soluble THTNB in a strongly alkaline (5 M NaOH) solution heated on a stream bath has been reported [17]; however, it has never been shown that TATB can slowly hydrolyze in water under ambient conditions. In this respect, 2,4,6-trinitroaniline has been reported to undergo slow hydrolysis in a moist environment to produce 2,4,6-trinitrophenol [18].

In addition to the formation of mono-benzofurazan (C1), the reduction of a nitro group to form 1,3,5-triamino-2-nitroso-4,6-dinitrobenzene (C4) also occurred during photolysis of TATB with UV light (Scheme 1, route 3). The production of the mono-nitroso analog (C4) of TATB through UV irradiation has also been reported by Manaa et al. [6]. Xiong et al. [8] suspected that the production of the mono-nitroso analog could be a minor photodegradation product of TATB. In our study, 1,3,5-triamino-2-nitroso-4,6-dinitrobenzene (C4) was also a minor degradation product. The denitration of solid TATB with ultraviolet light has been reported [19,20]. Although in very small amounts, the detection of compound C5 (1,3,5-triamino-2,4-dinitrobenzene) suggests that denitration of TATB also took place in solution irradiated at 254 nm. Compound C6 (mono-benzofuroxan) was only detected in the irradiated (254 nm)/sonicated (35 kHz) samples followed by a second irradiation step at 254 nm. It was previously established that when a mechanically sensitive chemical is exposed to ultrasound, a rearrangement occurs and yields products that cannot be obtained from purely thermal or light-induced reactions [21], which appears to be the case for one of the photolytic intermediate of TATB. Since C5 was only detected in trace amounts and C6 only formed under specific conditions (the combination of sonication and UV), their formation pathways are not discussed in detail here. While C-NO_2_, a key functional group in TATB, isomerization to C-ONO leading to the formation of ^•^NO free radical and the phenolic analog triamino-dinitrophenol was calculated to be energetically more favorable under photolysis [8] or thermolysis [14] than denitration, the calculated mass ion of the compound triamino-dinitrophenol, [M − H]^−^ *m*/*z* 228.0374 Da was not found in our TATB-photolyzed samples.

Interestingly, despite the presence of water, the hydrolysis product 3,5-diamino-2,4,6-trinitrophenol (C3) and its precursor, the *aci*-nitro isomer (C2), were not found in the samples photolyzed by UV light, suggesting that nitro-*aci*-nitro isomerization is only related to sunlight. 

Finally, it is highly probable that the main degradation product, mono-benzofurazan, detected in the two photo-systems was responsible for the green color of the photolyzed TATB.

Scheme 1 summarizes the potential initial routes involved in the photolysis of TATB particles presented in this study.

## 4. Materials and Methods

### 4.1. Chemicals 

The 1,3,5-Triamino-2,4,6-trinitrobenzene (TATB, MW 258.15), a yellow powder, was provided by Defence Research and Development Canada (DRDC)-Valcartier. The reference standard solution of TATB (40 mg L^−1^ in dimethylformamide (DMF)) was purchased from Chromatographic Specialties (Brockville, ON, Canada). The ^18^O-labelled water (98 atom % ^18^O) was obtained from Sigma-Aldrich (Oakville, ON, Canada).

### 4.2. Water Solubility Measurement

The TATB (8 mg) was stirred in water (80 mL) at pH 4.0, 8.3 and 10.4 at environmentally-relevant temperatures, 10, 25, and 30 °C, over 18 days and protected from light. An aliquot was analyzed daily for TATB using a Waters HPLC-UV system (Milford, MA, USA) fitted with a Discovery C18 column (250 × 4.6 mm, 5 µm) (Supelco, Oakville, ON, Canada) and a UV/Vis detector at 35 °C. An injection volume of 50 μL was used. The mobile phase (50% methanol: 50% water) was run isocratically at 1 mL min^−1^. The detector was set to scan from 192 to 450 nm. The samples were injected with 50% DMF in water. Quantification was performed at 355 nm, a wavelength at which DMF absorbed poorly. The instrumental detection limit was estimated to be 0.005 mg L^−1^ at 355 nm. The amount of TATB solubilized in the samples incubated over 18 days at 10 and 25 °C, independently of the pH (4.0, 8.3 and 10.4), was below the instrumental limit of detection of 0.005 mg L^−1^. TATB could only be detected at 30 °C at a concentration of 0.01 mg L^−1^.

### 4.3. Photolysis of TATB Particles Using Simulated Solar Light (SolSim) and UV at λ = 254 nm

In a typical experiment, a thin layer of TATB particles (2.5 ± 0.7 mg or 50 ± 5 mg) in a quartz crucible (Ø 25 mm) was photolyzed for 4 days using a SolSim solar simulator (Luzchem Research, Inc., Ottawa, ON, Canada). The total power of the solar simulator output spectrum was calibrated to the best approximation of ASTM Air Mass 1.5 Global Tilt Standard in the 280–800 nm regions: total irradiance of 590,000 mW m^−2^. For the extraction of the photolyzed samples, dimethylsulfoxide (DMSO) and acetonitrile were used as solvents to solubilize TATB. To enhance solubilization, the mixtures were vortexed or sonicated for 10 min at a frequency of 35 kHz (Aquasonic 150D, VWR Sci. Product). The solution was then filtered through a 0.45 μm Millipore Millex-HV syringe filter before LC-MS analysis. Some photolyzed samples were mixed with acetonitrile and H_2_^18^O (1:1 *v*/*v*) and stored for 1 h prior to analysis to monitor the incorporation of ^18^O into the hydrolysis products. 

In another experiment, the photolysis of TATB (10 ± 2 mg) in 5 mL of water or 50% acetonitrile (*v*/*v*) in water (MeCN-50) mixture placed in 20 mL quartz tubes was performed in a Rayonet photoreactor equipped with 16 UV (254 nm) lamps (Branford, CT) for 24 h. TATB in MeCN-50 (0.7 mg L^−1^) was prepared by sonication and also subjected to irradiation at λ = 254 nm for a total of 60 min. Dark controls protected with aluminum foil were found stable under otherwise similar conditions. The experiments were carried out in duplicates or triplicates.

### 4.4. Alkaline Hydrolysis of TATB

The TATB particles (8 ± 2 mg) were incubated at 25 °C and 50 °C under static conditions in 80 mL of alkaline solution (NaOH) at pH 13 and protected from light. A control containing TATB (8 ± 2 mg) in MilliQ water (pH 5.5) was also incubated at 50 °C. An aliquot was analyzed daily, then each week, for TATB using the HPLC method described above, for up to 9 months (aged samples). 

### 4.5. LC-MS Analysis

The degradation products were analyzed with a Bruker MicroTOF-Q mass analyzer connected to an Agilent HPLC/UV system equipped with a DAD detector. For mass analysis, electrospray ionization in the negative mode (ESI-) was used, mainly producing deprotonated molecular mass ions (M – H)^−^. The mass detector was set to scan in the range from 50 to 1000 Da. Samples (10 μL) were injected into a Gemini NX C18 column (2.1 mm × 150 mm, 3 micron particle size; Phenomenex) at 25 °C. The mobile phase was a mixture of acetonitrile and water running by gradient mode at a flow rate of 0.25 mL min^−1^. The samples were extracted and analyzed fresh, or after being stored at room temperature for several months (aged samples).

## 5. Conclusions

Extensive LC-MS analysis revealed that the distribution of the products of TATB photolyzed in a SolSim solar simulator as solid is slightly different from that obtained in aqueous or in a mixture of acetonitrile and water (1:1 *v*/*v*) solution of TATB using a Rayonet photoreactor at 254 nm. The main degradation product, mono-benzofurazan, seemingly in a green color formation, was observed in both photolytic systems. However, the nitro-*aci*-nitro isomer of TATB was only produced using simulated sunlight, and readily hydrolyzed to 3,5-diamino-2,4,6-trinitrophenol (C3), which is much more soluble in water than TATB. Product C3 was also detected in aqueous solutions of TATB aged under natural environmental conditions during thermolysis at 50 °C, or during alkaline hydrolysis at pH 13. 

The experimental evidence gathered in this study showed that the energetic chemical TATB can phototransform under simulated solar conditions and hydrolyze in aged aquatic systems to produce several intermediates, including polynitrophenol derivatives. This proven transformation of the insoluble TATB into apparently more soluble compounds is a cause of environmental concern due to potential migration leading to the contamination of groundwater, which may pose a risk to aquatic life.

## Figures and Tables

**Table 1:** TATB and photo-transformation products detected in this study. Compounds C4, C5, and C6 were only detected in samples irradiated with UV light.

ID	Name	*m*/*z* [M − H]^−^	MW g mol^−1^	Chemical Formula	Structure Proposed
TATB	1,3,5-triamino-2,4,6-trinitrobenzene	257.0394	258.15	C_6_H_6_N_6_O_6_	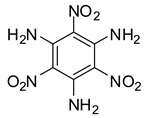
C1	mono-benzofurazan	239.0170	240.13	C_6_H_4_N_6_O_5_	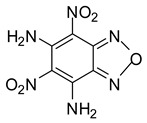
C2	*aci*-nitro isomer of TATB	257.0276	258.15	C_6_H_6_N_6_O_6_	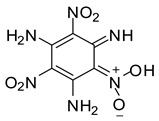
C3	3,5-diamino-2,4,6-trinitrophenol	258.0116	259.13	C_6_H_5_N_5_O_7_	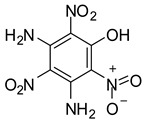
C4	1,3,5-triamino-2-nitroso-4,6-dinitrobenzene	241.0327	242.15	C_6_H_6_N_6_O_5_	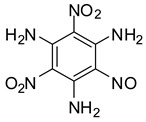
C5	1,3,5-triamino-2,4-dinitrobenzene	212.0425	213.15	C_6_H_7_N_5_O_4_	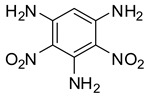
C6	mono-benzofuroxan	255.0120	256.13	C_6_H_4_N_6_O_6_	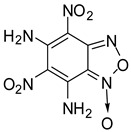

## Supplementary Materials

The following supporting information can be downloaded: Figure S1: UV and MS spectra of compound C1.

## Sample Availability

Not available.
