# Supplementary figures and images for: Photolysis of the Insensitive Explosive 1,3,5-Triamino-2,4,6-trinitrobenzene (TATB)
Source: Molecules. 2021 Dec 30;27(1):214. doi: 10.3390/molecules27010214 (PMC8746464; doi:10.3390/molecules27010214)

SUPPLEMENTARY FIGURE

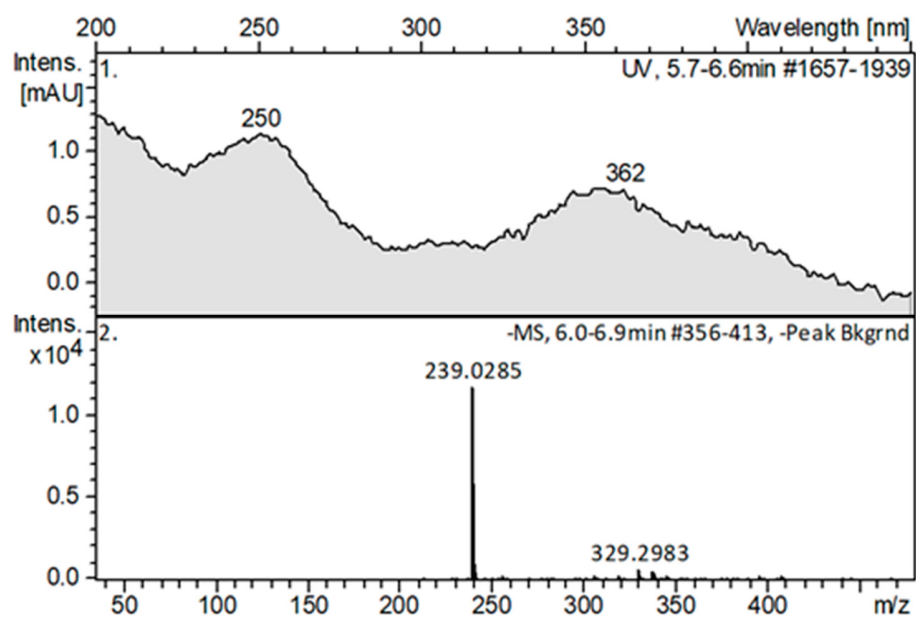

Figure S1. UV and MS spectra of compound C1

Supplement: Supplementary file 1 [file molecules-27-00214-s001.zip › molecules-1514225-supplementary.pdf]
